# L1 production following brief L2 exposure: Evidence for cross-talk across comprehension and production

**DOI:** 10.3758/s13423-024-02572-7

**Published:** 2024-09-16

**Authors:** Tamar Degani, Hamutal Kreiner, Mathieu Declerck

**Affiliations:** 1 Department of Communication Sciences & Disorders, University of Haifa, Mount Carmel, Haifa, Israel; 2 Ruppin Academic Center, Kfar Monash, Israel; 3 Vrije Universiteit Brussel, Brussels, Belgium

**Keywords:** Blocked-language order effect, Language comprehension, Language production, Language control, L2 after-effect

## Abstract

Language control allows bilinguals to fluently shift between their languages. Here, we tested whether comprehension and production tasks initiate language control processes to the same extent, and whether these processes operate over specific concepts or globally. Seventy Hebrew–English bilinguals completed an L1 picture-naming production task in the first and third blocks, and either a reading aloud (word production) or an animacy judgment (word comprehension) task in their L2 in the second block. Further, concepts were either repeated across blocks or not. Results showed more filled pauses in the third block relative to the first block. Additionally, the size of this blocked-language order effect was similar following word production and word comprehension tasks in the L2, suggesting that production and comprehension tasks were similarly efficient in instigating control processes. Finally, both recurring and new concepts were affected, suggesting that mostly global language control is at play. These findings provide constraining evidence for fully understanding the scope of bilingual language control.

**Supplementary Information:**

The online version contains supplementary material available at 10.3758/s13423-024-02572-7.

All languages known to a bilingual tend to be activated in parallel and compete with each other (e.g., Costa et al., 2000; Kroll et al., 2006). To alleviate this cross-language competition, processes of language control are involved. While one would assume that a similar language-control process is implemented during bilingual word production and bilingual word comprehension (e.g., Declerck & Grainger, 2017; Peeters et al., 2014; Silbert et al., 2014), not all evidence points in this direction (e.g., Ahn et al., 2020; Blanco-Elorrieta & Pylkkänen, 2016; Mosca & de Bot, 2017). Here, we investigate the possibility of shared language-control processes in word comprehension and word production and test whether they operate over specific representations only or affect the lexicon as a whole.

One prominent measure of language control is the blocked-language order effect (e.g., Declerck, 2020; Kreiner & Degani, 2015; Misra et al., 2012), in which bilinguals perform three single language blocks; Language A is used in Blocks 1 and 3, and Language B is used in Block 2. This setup generally leads to worse performance in Block 3 compared with Block 1 (see also L2 after-effect; e.g., Casado et al., 2022),

Different accounts were proposed to explain this effect (Kreiner & Degani, 2015; Van Assche et al., 2013; for a review, see Declerck & Koch, 2023). The *activation account* (Branzi et al., 2014, 2016; Wodniecka et al., 2020b) postulates that activation of Language B in Block 2 persists into Block 3, making Language B representations more effective competitors to Language A representations in Block 3. The *inhibition account* (e.g., Misra et al., 2012; Rossi et al., 2018, in line with the Inhibitory Control [IC] model, Green, 1998), suggests that producing Language B in Block 2 requires inhibition of Language A, which persists into Block 3, such that Language A is less available during Block 3 relative to Block 1. Wolna et al. (2024) recently suggested that *competition of nonlinguistic* mechanisms and the engagement of *domain-general cognitive control* are responsible for the observed effects.

Notably, whereas previous literature focused on Block 3 performance, here, we focus on Block 2—the phase at which presumably control processes are initiated. We ask whether control processes initiated during a comprehension task can affect subsequent production in a similar way as do control processes initiated during a production task.

## Language control in production and comprehension

The developmental version of the Bilingual Interactive Activation model (BIA-d; Grainger et al., 2010), which incorporates language inhibition via language nodes (rather than task schemas, as in the IC model), postulates that the main difference of production-based versus comprehension-based language control is how inhibition is initiated. In comprehension, bottom-up processing of words activates the language nodes, whereas in production, the goal to speak a specific language operates in a top-down fashion to activate language nodes. Critically, regardless of how inhibition is instigated, both comprehension and production should lead to inhibition exerted from the shared language nodes. Hence, both a comprehension task and a production task would influence subsequent performance in the other language.

In line with this prediction, Peeters et al. (2014) observed that French-English bilinguals named pictures in the L1 more slowly after making a lexical or semantic decision on an L2 written word than when no language switch occurred (see also Gambi & Hartsuiker, 2016). Li and Gollan (2022) observed that Spanish–English bilinguals were slower to name pictures in their dominant language following a semantic classification task on words in their nondominant language (but not following a read-aloud task), again suggesting that language control operated across comprehension and production. Of note, this line of research utilizes a paradigm where switching takes place on a trial-by-trial basis. However, the degree to which more sustained language control processes, tapped by the blocked-language order effect, are modulated by a shift between comprehension and production tasks, is not addressed in these studies (see Declerck, 2020, for differences between these paradigms).

More direct evidence for the possibility that comprehension tasks can instigate sustained language control processes comes from Kreiner and Degani (2015) and Stasenko and Gollan (2019). In these studies, bilinguals named pictures in their L2 in Block 1 and Block 3, while Block 2 included a comprehension task of watching a movie in the L1. More tip-of-the-tongue incidents in the L2 were observed in Block 3 than in Block 1. Hence, language control instigated during a *comprehension* block (watching a movie) affected subsequent *production*, suggesting cross-talk across comprehension and production.

However, there is also evidence against the claim made by the BIA-d (Grainger et al., 2010) that language control is shared across comprehension and production (e.g., Ahn et al., 2020; Declerck et al., 2019; Mosca & de Bot, 2017). For instance, while bilingual language production studies tend to find a blocked-language order effect (e.g., Branzi et al., 2014; Casado et al., 2022), there was no evidence for such a pattern when combining data from a series of three experiments with French–English and French–Spanish bilinguals and a separate experiment with French–English bilinguals when all single language blocks included comprehension tasks (Declerck et al., 2019, including magnitude, parity, animacy, and size tasks). This difference could be due to production being a more active process in which speakers need to select a single response among multiple candidates, whereas comprehension is a more passive process in which listeners need to tune their attention and monitor the features of the input (Blanco-Elorrieta & Pylkkänen, 2016). Critically, because production entails articulation of a single response, there is a greater pressure toward language selection (Kroll et al., 2008), which may recruit language control to a greater extent than in comprehension tasks.

Given the scarcity and inconclusive nature of available evidence, the main goal of the present project was to test whether language control is similarly instigated in language comprehension and production. To this end, Hebrew–English bilinguals named pictures in Hebrew in Block 1 and Block 3, and performed either a reading-aloud task or an animacy-judgment task in Block 2. These tasks serve as proxies of word production and word comprehension, respectively (see also Li & Gollan, 2022; see Table 1 for task subcomponents). The critical difference across these particular tasks is in phonological and phonetic encoding, articulation, and response output, as the lexical item is provided in both tasks in the form of a written word. This feature of our design allowed us to keep the stimuli identical across the two tasks (written words) in the exposure block.

**Table 1:** Subcomponents of the particular tasks used in the current study

Subcomponent	Input	Conceptual processing − Preparation /Access	Lexical selection	Morphological & phonological encoding	Phonetic encoding	Articulation	Response decision	Response output
Task								
Production task: Picture naming [Blocks 1 &3]	Picture	Yes	Yes	Yes	Yes	Yes	No	Oral production
Production task: Reading aloud [Block 2]	Written word	Not required	Provided	Yes	Yes	Yes	No	Oral production
Comprehension task: Animacy Judgment [Block 2]	Written word	Required after lexical access	Provided	No	No	No	Yes	Manual (pen-&-paper task)

Stages adapted from the theory of Levelt et al. (1992). Input, response decision, and response output were added to differentiate the three tasks

If only production tasks can initiate language control processes, then one would expect to find a blocked-language order effect when all blocks include word production, but not when the exposure Block 2 includes word comprehension. Conversely, if both production and comprehension can lead to the engagement of similar language-control processes, a blocked-language order effect should emerge when comprehension is used in the intervening Block 2.

## Global and local language control

An additional goal was to investigate whether language control influences the entire language (i.e., global control) and/or whether it influences specific target words (i.e., local control). Language control may operate via a language schema/node, affecting all representations of a given language, or via influence on language lemmas, thus affecting specific items (Declerck & Philipp, 2017).

To tap local language control, studies have typically utilized repeated stimuli across language blocks (e.g., Guo et al., 2011; Misra et al., 2012). A change in language from one block to the next resulted in reduced stimulus repetition facilitation, taken to index local language control. Conversely, to test global language control studies utilized different stimuli in Block 2 and Block 3 (e.g., Casado et al., 2022; Kreiner & Degani, 2015; Stasenko & Gollan, 2019). Branzi et al. (2014) and Wodniecka et al. (2020b) included both repeated and new items, and observed no additional effect for repeated items beyond the global control processes observed for nonrepeated items. However, as in such designs language control is measured by different indices for repeated and new items (reduction in stimulus repetition facilitation for local control versus worse performance for global control), direct comparisons between the two processes are difficult to make. Degani et al. (2020) alleviated this issue by repeating the same concepts, rather than the exact same words or pictures, across blocks. Arabic–Hebrew bilinguals named pictures in Arabic in Block 1 and Block 3, but read Hebrew words out loud in Block 2. Half the concepts used in the pictures of Block 3 were also used as written words in Block 2. Higher error rates in Block 3 relative to Block 1 were observed for both repeated and new concepts, but more cross-language errors were found with repeated concepts. Thus, language control might operate not only at the global level, but local effects may be reflected in some measures but not others.

In the current study, we used a similar setup as Degani et al. (2020). If there is local language control, one would expect a larger blocked-language order effect on repeated concepts, since language control is implemented twice (at the local and the global level), whereas new concepts will only be influenced by language control at the global level. Critically, here, we also test this effect with a comprehension exposure task, focusing on error rates and filled pauses as our dependent measures.^1^

## Methods

### Participants

Seventy Hebrew–English bilingual participants (18 men; 52 women), who were students at a Hebrew-speaking University in Israel at the time of testing, were recruited. All were native Hebrew speakers, who began learning English during elementary school and consider themselves moderately proficient in English. All had normal hearing and vision, and reported no learning disabilities. Participant characteristics based on a language history questionnaire administered at the end of the experimental tasks (adapted from the Leap-Q; Marian et al., 2007) are summarized in Table 2. Comparisons across groups revealed that although the two groups were sampled from the same population, there were significant differences between the two groups in self-reported English L2 age of acquisition, English talking proficiency, and exposure to the L1 during listening. These dimensions were therefore considered statistically in the analyses. The data of four additional participants were excluded from analysis (one due to a technical problem during administration, two because of a learning disability, and one because he was a native speaker of another language).

**Table 2:** Participant characteristics as a function of exposure type

Measure	Exposure Block 2	
	Animacy judgment Word comprehension	Reading aloud Word production
Number of participants	35	35
Gender	11 men; 24 women	7 men; 28 women
Age (years)	26.5 (2.8)	24.9 (4.1)
Formal education (years)	14.8 (2.1)	13.9 (2.2)
Formal education of mother (years)	14.1 (2.1)	14.5 (3.6)
Age began learning L2 (years)*	8.2 (1.9)	7.1 (2.1)
Hebrew reading proficiency^a^	9.5 (0.7)	9.7 (0.5)
Hebrew writing proficiency^a^	9.5 (0.7)	9.5 (0.7)
Hebrew talking proficiency^a^	9.8 (0.5)	9.5 (0.7)
Hebrew understanding proficiency^a^	9.9 (0.4)	9.7 (0.5)
English reading proficiency^a^	7.7 (1.0)	7.7 (1.6)
English writing proficiency^a^	6.4 (1.5)	7.0 (1.8)
English talking proficiency^a^ *	8.3 (0.8)	7.5 (1.7)
English understanding proficiency^a^	8.5 (0.7)	8.1 (1.4)
Hebrew reading use^b^	9.3 (1.0)	9.2 (1.2)
Hebrew writing use^b^	9.6 (0.6)	9.5 (0.7)
Hebrew talking use^b^	9.8 (0.4)	9.8 (0.4)
Hebrew listening use^b^ *	7.7 (1.9)	6.5 (2.7)
English reading use^b^	7.4 (2.0)	6.9 (2.0)
English writing use^b^	4.4 (2.3)	4.1 (2.3)
English talking use^b^	4.3 (2.4)	4.3 (2.5)
English listening use^b^	7.7 (1.5)	7.9 (2.1)

^a^ Self-rated scores on a scale of 0 (low proficiency) to 10 (high proficiency)

^b^ Self-rated scores on a scale of 0 (*low use*) to 10 (*high use*)

* a significant difference based on an independent-samples *t* test with a *p* < .05

### Stimuli

A set of 100 colored pictures were selected from a previous norming study with native Hebrew speakers (Hirosh et al., 2024). The set included pictures from the Moreno-Martínez and Montoro’s (2012) stimulus database, as well as from other freely available online sources, all of which corresponded to noncognate words across Hebrew and English as determined by two Hebrew–English bilinguals. The selected set included two lists of 50 pictures each, matched on name agreement, visual complexity, familiarity of the object, typicality of the picture, length in number of syllables and number of letters, and word frequency in Hebrew (based on HebWaC corpus via SketchEngine; see Kilgarriff et al., 2010, 2014; all *t* values < 1). Each list was divided into two matched subsets such that half of the pictures in each subset (*n* = 25) were included in the exposure block (i.e., repeated items), and half were not. New and repeated items were carefully matched but were not fully rotated across participants. Specifically, repeated and new items were matched within each list on name agreement, familiarity of the object, typicality of the picture, length in number of syllables in Hebrew and in number of letters, and on word frequency (all *p* values > .13). However, repeated items were found to have a slightly higher visual complexity compared to new items (*p* = .014; see Table 3). Visual complexity was therefore considered as a covariate in the analysis. The stimuli list is available in the Appendix.

**Table 3:** Mean item characteristics (*SD* in brackets) as a function of repetition

Measure	Repeated words	New words	All words
*N*	50	50	100
Hebrew length (in letters)	4.5 (1.1)	4.2 (1.3)	4.4 (1.2)
Hebrew length (in syllables)	2.4 (.73)	2.3 (.84)	2.4 (.78)
Picture name agreement	.96 (.03)	.96 (.04)	.96 (.03)
Picture visual complexity *	2.1 (.44)	1.9 (.37)	1.96 (.42)
Object familiarity	6.3 (.42)	6.3 (.37)	6.3 (.39)
Object typicality	6.0 (.50)	6.1 (.44)	6.0 (.47)
Log written word frequency	.60 (.60)	.72 (.64)	.66 (.62)

^*^ marks a significant difference at *p* < .05

### Design and procedure

**Fig. 1:**
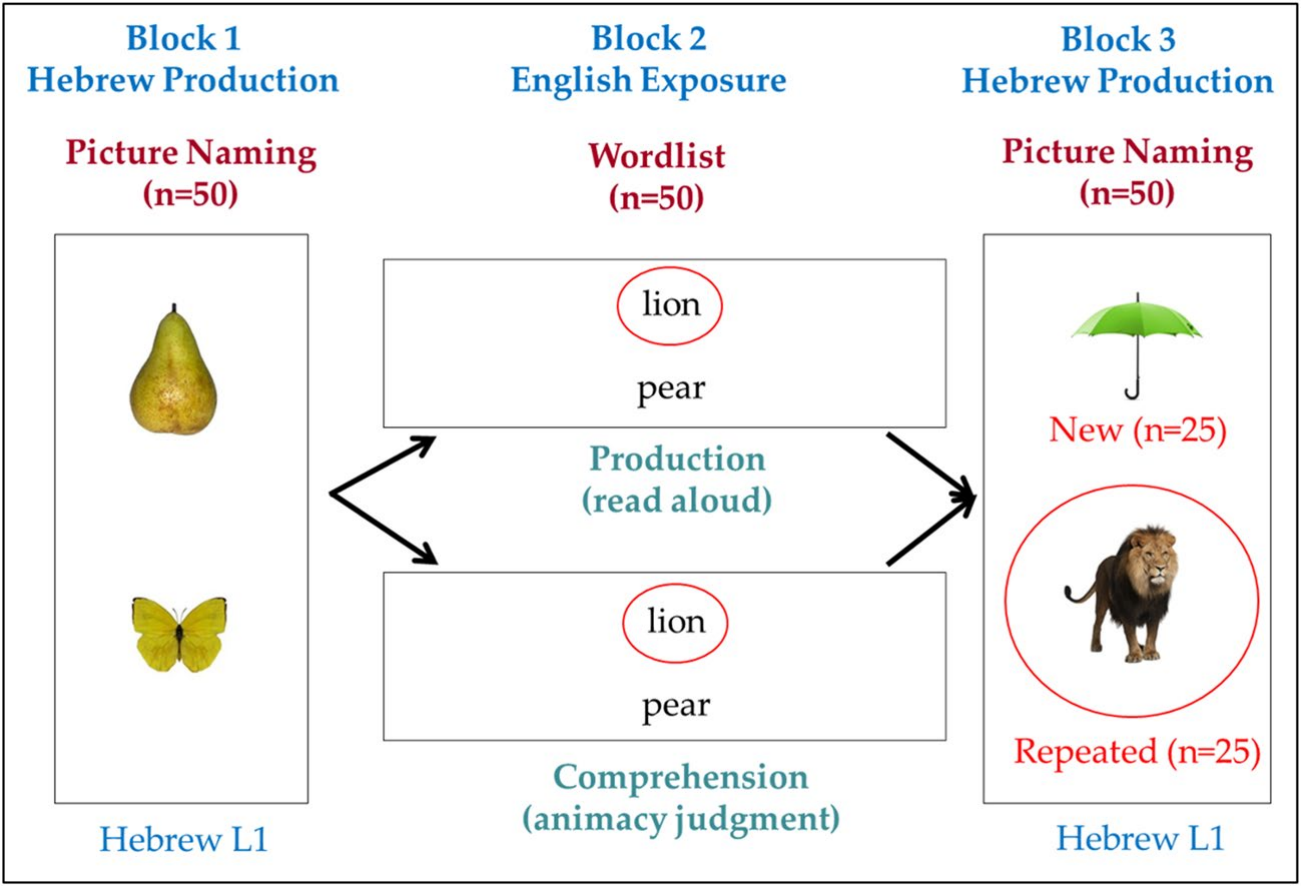
Overall design of the study. Participants completed all 3 blocks. Blocks 1 and 3 included pictures to be named in Hebrew, whereas Block 2 included English written words, on which participants performed either a reading aloud or an animacy judgment task. Concepts in Block 3 were either repeated from Block 2 (e.g., lion) or were completely new. (Color figure online)

Each participant was tested individually in a quiet room, and all communication with the experimenter prior to the experimental tasks was conducted in Hebrew. Participants completed a preexposure production task in L1 Hebrew (Block 1) on a set of 50 pictures, presented one at a time, then an English exposure task (Block 2) on a set of 50 written English words (presented together as a list), and finally a postexposure production task in L1 Hebrew again (Block 3) on a different set of 50 pictures (again presented one at a time; see Fig. 1). Picture list order across Block 1 and Block 3 was counterbalanced across participants, and the order of pictures within each list was initially randomized and then kept constant for all participants. In Blocks 1 and 3, participants were asked to name each picture as quickly and accurately as possible in Hebrew. Responses were recorded for later coding of accuracy and the presence of filled pauses. In exposure Block 2, half of the participants were asked to make an animacy judgment (yes/no, word comprehension task), and the other half a reading-aloud (word production) task. Both tasks were performed on the same set of 50 printed English words, half of which corresponded to pictures presented in Block 1, and the other half corresponded to pictures to be presented in Block 3. As a result, in Block 3, pictures could refer to a repeated concept from Block 2 (where it was represented by a written word) or be a completely new item, not appearing in either Block 1 or 2.

Our focus was on error rates and filled pauses, as these have been shown to increase as lexical selection becomes more difficult (Hartsuiker & Notebaert, 2010; see also Declerck et al., 2021; and Sánchez et al., 2022, in the context of bilingual language control). Filled pauses (vocalizations that allow the speaker to fill in a gap during speaking, e.g., “hu,” “um,” and “er”) occur in natural speech, and may reveal processing difficulty, especially in production of words in isolation by nonnative speakers (Kosmala & Crible, 2022). Moreover, whereas response-time (RT) analyses require exclusion of trials on which the voice key is triggered by anything other than the initial phoneme of the target word (including expressions such as “ah” or “um,” which is not an error and not a technical problem), filled pauses may be more informative about processing difficulty with more variable accuracy rates, as when using less-frequent items. In such cases, accuracy rates may not be sufficiently high to support stable RT analysis (Bruyer & Brysbaert, 2011). RTs were therefore not recorded in the current study.

### Analyses

Data are available on the OSF platform (https://osf.io/tz76u/?view_only=73c8575f0b4a41e38b7b500d0fe619e2). Recorded responses were transcribed and coded by a native Hebrew speaker. A response was considered an error when a wrong concept was named in the correct language, a correct concept was given in the wrong language, no answer was given, or when the participant indicated not knowing the correct response. Our analyses focused on filled pauses, as these are known to be a measure of increased cognitive load (Hartsuiker & Notebaert, 2010; Sugiura et al., 2020), and have been used as such in previous language control studies (Declerck et al., 2021; Sánchez et al., 2022), but are novel in the context of the blocked-language order effect. The filled pauses measure was binary, with 1 indicating trials on which hesitations with any vocalizations that allow the speaker to fill a gap during speaking (e.g., “uh,” “um,” “er”) were made.

The errors and filled pauses were analyzed using logistic mixed models. Both participants and items were considered random factors with all fixed effects and their interactions varying by all random factors (Barr et al., 2013).^2^ Block (first block = −0.5; third block = +0.5), stimulus repetition (repeated = −0.5; new item = +0.5), exposure type (comprehension = −0.5; production = +0.5) and their interactions were the relevant fixed factors.

## Results

**Fig. 2:**
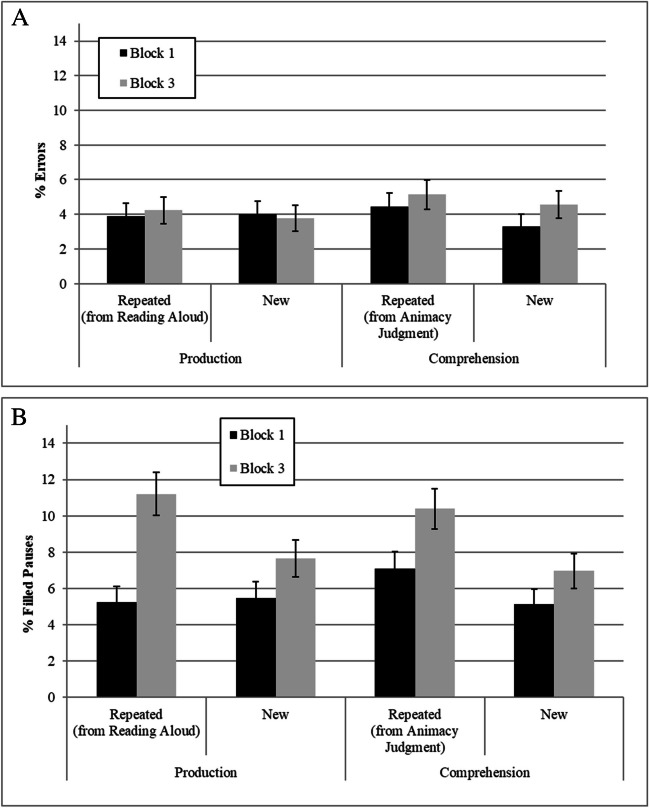
Error rates (**A**) and filled pauses (**B**) as a function of exposure type, block, and stimulus repetition. Error bars represent standard errors calculated for within-participant variables (following Morey, 2008)

Error rates were relatively low (less than 5%; see Fig. 2a), with no significant main effects or interactions (all *F* values < 1). However, analysis of the filled pauses data showed a significant effect of Block (*b* = 0.52, *SE* = 0.21, *z* = 2.48, *p* = .013), with more filled pauses occurring in Block 3 (9.1%) than in Block 1 (5.7%), and a marginally significant effect of stimulus repetition (*b* = 0.49, *SE* = 0.25, *z* = 1.93, *p* = .054), with more filled pauses occurring when repeated items were produced (8.5%) than when new items were produced (6.3%). There was no interaction between block and stimulus repetition, and, critically, no significant effect or interaction with exposure type (all *p* values > .36).^3^

Due to our theoretical interest, and to verify that the block effect is not driven by the production exposure condition only, we examined whether the block effect was significant in each exposure type using Bonferroni corrections for multiple comparisons. Results showed a significant block effect both when exposure required word production (value = 0.34, χ^2^ = 11.60, *p* = .001) and when it entailed a word-comprehension task (value = 0.37, χ^2^ = 6.16, *p* = .026; see Fig. 2).

Further, because new and repeated items were carefully matched but were not fully rotated across participants, we examined the block effect separately for each stimulus repetition level. The block effect was significant for repeated items (6.2% to 10.8%, value = 0.33, χ^2^ = 11.25, *p* = .002), but only marginally significant for new items (5.3% to 7.3%, value = 0.38, χ^2^ = 4.45, *p* = .069), suggesting a stronger effect for repeated items.

## Discussion

We observed a similar, significant blocked-language order effect, when the intervening exposure Block 2 included comprehension and when it included production. Further, both recurring concepts and new concepts were produced less efficiently, with more filled pauses following exposure to the other language, although the effect appeared stronger for repeated concepts. We address these issues below.

### Language control across comprehension and production

Transition from one language to the other resulted in a cost, as L1 production performance was hindered following brief exposure to the L2. This pattern is consistent with the literature on the blocked-language order effect (e.g., Branzi et al., 2014; for a review, see Declerck, 2020), and suggests that performance in the dominant language is affected by exposure and previous engagement with stimuli in the nondominant language.

Critically, the current study provides no evidence for differential language control mechanisms that are instigated during comprehension and production. More filled pauses during production were observed following a word comprehension task in the other language compared with performance prior to L2 exposure, suggesting cross-talk across comprehension and production (see also Kreiner & Degani, 2015; Stasenko & Gollan, 2019). Extending past research, language control was evident here following a lexical comprehension task on production of both repeated and new items.

The mere change from comprehension to production cannot serve as a viable explanation for the emergence of the blocked-language order effect because the size of the effect was similar when Block 2 entailed word comprehension relative to when it entailed word production. Further, one may argue that a task change may explain the observed effects. Li and Gollan (2022) reported switch cost effects in picture naming following a semantic decision task but not following a reading-aloud task, hypothesizing that switching between less similar tasks would result in greater costs than switching between more similar tasks. Because the current study utilized the same tasks as in Li and Gollan (2022), if the degree of task similarity was responsible for the effects, we should have observed larger blocked-language order effects when shifting from animacy judgment to picture naming (less similar) than when shifting from reading aloud to picture naming (more similar; see Table 1). Contrary to this prediction, however, we observed similar-sized effects following both tasks. Thus, trial-by-trial language control indexed by language switching studies (as measured by Li & Gollan, 2022) may differ from sustained language control tapped here by the blocked-language order effect (cf. Declerck & Koch, 2023).

Three explanations can account for the blocked-language order effect observed here. First, the pattern is consistent with the predictions of the BIA-d model (Grainger et al., 2010), by which shared language nodes across comprehension and production inhibit nontarget language representations. L2 exposure in Block 2, regardless of whether it entailed comprehension or production, resulted in inhibition on L1 which consequently required recovery from inhibition and lead to reduced accessibility in Block 3.

Second, the effect may also be explained within an activation-based account (e.g., Branzi et al., 2014; Wodniecka et al., 2020b). Language use, through either production or comprehension, resulted in activation of the relevant representations. On the subsequent block, these nontarget language representations were highly active, regardless of what caused their increased activation, and served as more effective competitors, hindering performance in Block 3 relative to Block 1. Comprehension (an animacy judgment task) and production (reading aloud written words) lead to a similar-sized boost in activation to language representations during Block 2.

Finally, Wolna et al. (2024) utilized fMRI to trace the neural basis of bilingual language control in a related setup (L1 production after an L1 or an L2 block). Their findings did not support the presence of competition at the lexical level, and instead lead to the proposal that the findings reflect either interference at a non-linguistic task schema level, or is linked to the engagement of domain-general control mechanisms. Our behavioral findings would suggest that under this account, as well, both comprehension and production processes lead to similar task-schema competition or engagement of domain-general control.

The current study does not allow one to distinguish between these three explanations, but careful consideration of the task components utilized here allows one to draw constraining evidence to be incorporated in future modeling of bilingual language control. Specifically, as seen in Table 1, the critical difference between these exposure tasks lies in the response output and the associated encodings that precede it, but in both tasks the lexical item was provided in the form of a written word. Thus, engagement of sustained language control does not require that participants start from conceptualization. Similarly, there is no need to select a single response for overt speech production, as one is not needed in the animacy judgment task. Indeed, articulation does not seem to be a prerequisite for language control processes to be engaged (see also Kreiner & Degani, 2015; Stasenko & Gollan, 2019). Conversely, a response-decision component being present in the animacy judgment task is not required, because reading aloud did not entail such a phase (see also previous work by Degani et al., 2020; Kreiner & Degani, 2015). Taken together, the findings suggest that the mere engagement with linguistic stimuli (i.e., mere activation) is sufficient to initiate the cascaded language control processes that result in performance decrements on the subsequent block in the other language.

The blocked-language order effect observed when the intervening block entailed comprehension may be considered at odds with the findings of Declerck et al. (2019), who did not find an effect with comprehension tasks. However, in that study all three blocks included comprehension tasks (including an animacy task as used here), whereas here Block 1 and Block 3 included a production task. Together, this could suggest that both production and comprehension initiate comparable language-control processes, but that the consequences of this language control may be more readily detected via a production task. Thus, we hold that comprehension can instigate language control processes. Future studies in which production and comprehension are manipulated across all three blocks are needed.

It is possible that language control could be instigated more strongly in a production task that includes conceptualization, and it is also not clear whether all comprehension tasks, such as tasks that do not require deep conceptual processing or do not entail a response decision, will behave similarly to the animacy judgment used here (though no differences across comprehension tasks were observed in Declerck et al., 2019). Further, the similar sized effect observed here following word production or word comprehension may be sustained by different processes (Blanco-Elorrieta & Pylkkänen, 2016), which nonetheless result in the same behavioral pattern. Therefore, more research is needed to test the generalizability of the findings with other comprehension and production tasks, but language control models should differentiate what initiates the control process (here, in the exposure Block 2) from the consequences of this process (here, Block 3 performance).

### Global and local language control

The blocked-language order effect was not significantly modulated by concept repetition, consistent with previous studies which did not observe stronger effects for repeated items (e.g., Branzi et al., 2014). Such global language control has been suggested to be restricted to bilinguals with two dissimilar languages (Van Assche et al., 2013), or to bilinguals who need to shift regularly between their languages (Degani et al., 2020), but the current study demonstrates these global effects with moderately proficient bilinguals who are immersed in their L1 and do not switch between their languages often (as in Casado et al., 2022; for review of different populations tested, see Wodniecka et al., 2020a). Further, this global effect is documented here in the presence of a shift from comprehension to production.

The current study focused on errors and filled pauses, rather than RTs, because these measures have been successfully used in previous language control studies (Declerck et al., 2021; Sánchez et al., 2022), and we predicted lower accuracy rates for this stimulus set. Contrary to our expectation and the pattern observed in previous work (e.g., Degani et al., 2020), accuracy rates were at ceiling (possibly due to a different stimulus composition) and were thus a less sensitive measure. Critically, filled pauses revealed increased difficulty in producing dominant language words after brief exposure to the nondominant language. As such, our findings converge with those revealed by other measures, including RTs (e.g., Casado et al., 2022), fluency (Van Assche et al., 2013), tip-of-the-tongue (Kreiner & Degani, 2015; Stasenko & Gollan, 2019), cross-language errors (Degani et al., 2020), ERPs (Misra et al., 2012; Rossi et al., 2018; Wodniecka et al., 2020b) and fMRI (Guo et al., 2011; Wolna et al., 2024), underscoring the presence of these sustained global language control effects, and suggesting that special emphasis needs to be given to the preceding language context in which bilingual performance is evaluated.

## Conclusion

The current study demonstrates that language control processes should be studied in two complementary ways: one that examines what instigates control processes and one that examines the consequences of this engagement. Whereas previous work mostly focused on the outcome of control engagement, the current study focused on the phase of control initiation. An integration of our findings with the extant literature suggests that both comprehension and production processes can lead to engagement of control processes, but that production tasks are more sensitive as a measure of control outcome. Critically, for control processes to be initiated, the mere engagement with language representations is sufficient. Finally, the findings suggest that global control drives the effect with only minor contribution of item-based mechanisms.

## Supplementary Information

Below is the link to the electronic supplementary material.

Supplementary file1 (DOCX 36 KB)

## Data Availability

Materials are provided in the Appendix. All data are available online (https://osf.io/tz76u/?view_only=73c8575f0b4a41e38b7b500d0fe619e2). The experiment was not preregistered.

## Footnotes

1 Reaction times (RTs) were not recorded in the current study (see also Declerck et al., 2021; Degani et al., 2020; Kreiner & Degani, 2015; Sánchez et al., 2022; Stasenko & Gollan, 2019).

2 The following strategy was used in case of convergence issues with the fully randomized model (cf. Barr et al., 2013; Matuschek et al., 2017): First, random effects for the item-specific random slopes were excluded, starting with the higher-order interactions. If the issue was not resolved, the higher-order interactions of the participant-specific random slopes were excluded. If this did not resolve the issue, the lower-order terms were removed, again starting with the item-specific random slopes before moving on to the participant-specific random slopes.

3 Additional analyses were conducted on the filled pauses and error data using the same analyses, but including the covariate visual complexity, L1 listening use, English spoken proficiency, or L2 age of acquisition. The results showed the same pattern of significance as in the main analyses without these covariates, except for the filled pauses analysis with English talking proficiency as a covariate, in which the marginally significant stimulus-repetition effect became significant (*p* = .047).

## Code availability

Associated scripts are available online (https://osf.io/tz76u/?view_only=73c8575f0b4a41e38b7b500d0fe619e2).
