# Supplementary material for: L1 production following brief L2 exposure: Evidence for cross-talk across comprehension and production
Source: Psychon Bull Rev. 2024 Sep 16;32(2):749–59. doi: 10.3758/s13423-024-02572-7 (PMC12000200; doi:10.3758/s13423-024-02572-7)
Supplement: Supplementary file 1 — Supplementary file1 (DOCX 36 KB) [file 13423_2024_2572_MOESM1_ESM.docx]

***Appendix*** *- Stimuli List Characteristics*

| ***Set 1*** | | | | | | | | |
| --- | --- | --- | --- | --- | --- | --- | --- | --- |
| *Block 3* | *Hebrew Word* | *English Word* | *Hebrew Freq.* | *Hebrew Length (Syll)* | *Hebrew Length (Letters)* | *Hebrew Name Agreement* | *Visual Complexity* | *Familiarity* |
| Repeated | קשת | bow | 59.39 | 2 | 3 | 0.93 | 1.79 | 5.98 |
| Repeated | קרנף | rhino | 0.72 | 2 | 4 | 1.00 | 2.05 | 6.24 |
| Repeated | טבעת | ring | 12.34 | 3 | 4 | 1.00 | 2.13 | 5.84 |
| Repeated | כוכב ים | starfish | 0.24 | 3 | 6 | 0.97 | 1.94 | 5.55 |
| Repeated | מסננת | strainer | 2.69 | 4 | 5 | 1.00 | 1.63 | 6.44 |
| Repeated | כינור | violin | 2.66 | 2 | 5 | 0.90 | 2.13 | 5.94 |
| Repeated | הר געש | volcano | 2.69 | 3 | 5 | 0.93 | 2.23 | 5.73 |
| Repeated | סירה | boat | 7.37 | 2 | 4 | 0.88 | 1.85 | 5.50 |
| Repeated | אצבע | finger | 22.11 | 2 | 4 | 0.94 | 1.84 | 6.26 |
| Repeated | זבוב | fly | 3.11 | 1 | 4 | 0.94 | 2.94 | 6.47 |
| Repeated | פטיש | hammer | 4.48 | 2 | 4 | 0.97 | 1.68 | 6.00 |
| Repeated | קרחון | iceberg | 2.24 | 2 | 5 | 0.97 | 2.80 | 5.27 |
| Repeated | צמה | braid | 1.19 | 2 | 3 | 1.00 | 2.87 | 6.50 |
| Repeated | פסנתר | piano | 6.97 | 2 | 5 | 0.97 | 2.13 | 6.52 |
| Repeated | קולב | hanger | 0.72 | 2 | 4 | 0.91 | 1.52 | 6.76 |
| Repeated | אופניים | bicycle | 44.21 | 4 | 7 | 0.90 | 2.52 | 6.68 |
| Repeated | חבית | barrel | 4.23 | 2 | 4 | 0.94 | 1.76 | 6.12 |
| Repeated | כפתור | button | 22.44 | 2 | 5 | 0.97 | 1.56 | 6.72 |
| Repeated | מטריה | umbrella | 1.95 | 3 | 5 | 0.97 | 1.97 | 6.93 |
| Repeated | דלעת | pumpkin | 3.06 | 2 | 4 | 0.97 | 1.85 | 6.24 |
| Repeated | שועל | fox | 5.47 | 2 | 4 | 0.93 | 2.67 | 6.23 |
| Repeated | מנעול | lock | 2.86 | 2 | 5 | 0.97 | 2.00 | 6.83 |
| Repeated | נדנדה | swing | 1.45 | 3 | 5 | 0.97 | 2.29 | 6.66 |
| Repeated | פטריה | mushroom | 1.65 | 3 | 5 | 1.00 | 2.03 | 6.73 |
| Repeated | כרית | pillow | 5.12 | 2 | 4 | 0.91 | 1.39 | 6.79 |
| New | עפרון | pencil | 2.42 | 3 | 5 | 1.00 | 1.22 | 6.59 |
| New | אגס | pear | 3.63 | 2 | 3 | 1.00 | 1.50 | 6.33 |
| New | יונה | pigeon | 27.3 | 2 | 4 | 0.90 | 2.00 | 6.29 |
| New | דג | fish | 88.77 | 1 | 2 | 1.00 | 1.97 | 5.71 |
| New | אקדח | revolver | 7.99 | 2 | 4 | 0.94 | 1.77 | 5.81 |
| New | חותמת | rubber stamp | 8.48 | 3 | 5 | 1.00 | 1.90 | 5.57 |
| New | ים | sea | 120.10 | 1 | 2 | 0.97 | 1.80 | 6.07 |
| New | עגבניה | tomato | 1.71 | 4 | 6 | 1.00 | 1.17 | 6.50 |
| New | מכנסיים | trousers | 7.79 | 4 | 7 | 0.90 | 1.10 | 6.23 |
| New | קסדה | helmet | 3.59 | 2 | 4 | 0.91 | 1.88 | 5.88 |
| New | פוני | bangs | 1.58 | 2 | 4 | 0.92 | 2.27 | 6.54 |
| New | חצוצרה | bugle | 1.13 | 3 | 6 | 0.97 | 2.03 | 5.38 |
| New | ענן | cloud | 19.72 | 2 | 3 | 0.93 | 2.57 | 6.63 |
| *Block 3* | *Hebrew Word* | *English Word* | *Hebrew Freq.* | *Hebrew Length (Syll)* | *Hebrew Length (Letters)* | *Hebrew Name Agreement* | *Visual Complexity* | *Familiarity* |
| New | פרה | cow | 13.09 | 2 | 3 | 1.00 | 1.80 | 6.70 |
| New | חציל | eggplant | 1.98 | 2 | 4 | 1.00 | 1.53 | 6.53 |
| New | מחבת | frying pan | 2.60 | 2 | 4 | 1.00 | 1.67 | 6.67 |
| New | שמלה | dress | 11.75 | 2 | 4 | 0.91 | 1.85 | 6.73 |
| New | מקטרת | pipe | 1.15 | 3 | 5 | 0.97 | 1.78 | 6.11 |
| New | אריה | lion | 41.69 | 3 | 4 | 1.00 | 2.56 | 6.81 |
| New | כבשה | sheep | 5.73 | 2 | 4 | 0.91 | 2.14 | 6.34 |
| New | מאפרה | ashtray | 0.37 | 3 | 5 | 0.94 | 1.85 | 6.38 |
| New | ברבור | swan | 1.50 | 2 | 5 | 0.92 | 2.38 | 6.54 |
| New | מספריים | scissors | 3.21 | 4 | 7 | 1.00 | 1.52 | 6.91 |
| New | מערוך | rolling pin | 0.55 | 3 | 5 | 0.94 | 1.47 | 6.69 |
| New | מקרר | refrigerator | 6.71 | 3 | 4 | 0.97 | 1.68 | 6.89 |
| ***Set 2*** | | | | | | | | |
| Repeated | טחנת רוח | windmill | 0.11 | 4 | 7 | 0.93 | 2.87 | 5.80 |
| Repeated | אופנוע | motorbike | 8.39 | 2 | 6 | 1.00 | 2.13 | 5.83 |
| Repeated | סיר | pot | 8.61 | 1 | 3 | 0.93 | 1.30 | 6.20 |
| Repeated | נחש | snake | 11.59 | 2 | 3 | 1.00 | 2.00 | 5.77 |
| Repeated | חצאית | skirt | 4.52 | 3 | 5 | 0.97 | 1.35 | 6.26 |
| Repeated | חרב | sword | 14.98 | 2 | 3 | 0.97 | 1.57 | 5.93 |
| Repeated | רכבת | train | 28.24 | 3 | 4 | 0.92 | 2.22 | 6.17 |
| Repeated | שקדים | almonds | 7.15 | 2 | 5 | 0.93 | 2.00 | 6.47 |
| Repeated | עצם | bone | 97.27 | 2 | 3 | 0.94 | 1.78 | 5.81 |
| Repeated | תנין | crocodile | 1.96 | 2 | 4 | 1.00 | 2.59 | 5.91 |
| Repeated | מלפפון | cucumber | 2.67 | 4 | 6 | 0.94 | 2.16 | 6.06 |
| Repeated | עט | pen | 5.79 | 1 | 2 | 0.97 | 1.73 | 6.73 |
| Repeated | כלה | bride | 18.63 | 2 | 3 | 1.00 | 2.87 | 6.53 |
| Repeated | מחשבון | calculator | 4.97 | 3 | 6 | 0.91 | 2.21 | 6.85 |
| Repeated | פעמון | bell | 3.04 | 3 | 5 | 0.97 | 2.26 | 6.23 |
| Repeated | עוגן | anchor | 5.97 | 2 | 4 | 1.00 | 2.35 | 6.35 |
| Repeated | עניבה | tie | 1.35 | 3 | 5 | 0.97 | 1.49 | 6.65 |
| Repeated | עפיפון | kite | 0.54 | 3 | 6 | 0.97 | 2.09 | 6.65 |
| Repeated | ידית | handle | 4.40 | 2 | 4 | 0.94 | 2.03 | 6.63 |
| Repeated | כפית | teaspoon | 11.60 | 2 | 4 | 0.94 | 1.53 | 6.88 |
| Repeated | ליצן | clown | 2.89 | 2 | 4 | 1.00 | 2.94 | 6.59 |
| Repeated | מגהץ | iron | 0.66 | 2 | 4 | 0.94 | 2.29 | 6.71 |
| Repeated | מכחול | paintbrush | 2.44 | 2 | 5 | 0.94 | 1.69 | 6.75 |
| Repeated | משאית | truck | 8.86 | 3 | 5 | 1.00 | 2.31 | 6.54 |
| Repeated | משרוקית | whistle | 0.25 | 3 | 7 | 0.97 | 1.61 | 6.94 |
| New | ביצה | egg | 12.34 | 2 | 4 | 0.94 | 1.72 | 6.66 |
| New | תפוז | orange | 6.24 | 2 | 4 | 1.00 | 1.13 | 6.23 |
| *Block 3* | *Hebrew Word* | *English Word* | *Hebrew Freq.* | *Hebrew Length (Syll)* | *Hebrew Length (Letters)* | *Hebrew Name Agreement* | *Visual Complexity* | *Familiarity* |
| New | מטוס | plane | 18.78 | 2 | 4 | 0.92 | 1.89 | 6.49 |
| New | רימון | pomegranate | 8.56 | 2 | 5 | 1.00 | 1.30 | 6.47 |
| New | תפוח אדמה | potato | 7.78 | 5 | 8 | 1.00 | 1.46 | 6.40 |
| New | תרנגול | rooster | 2.88 | 3 | 6 | 1.00 | 1.57 | 6.30 |
| New | צב | turtle | 3.65 | 1 | 2 | 0.97 | 1.97 | 6.10 |
| New | דובדבן | cherry | 2.18 | 3 | 6 | 0.94 | 1.56 | 6.47 |
| New | נמלה | ant | 1.62 | 3 | 4 | 0.97 | 1.97 | 6.50 |
| New | תפוח | apple | 11.40 | 2 | 4 | 0.94 | 1.91 | 6.50 |
| New | חץ | arrow | 6.42 | 1 | 2 | 0.94 | 2.00 | 5.65 |
| New | גרזן | axe | 1.41 | 2 | 4 | 0.94 | 2.06 | 5.39 |
| New | עטלף | bat | 0.85 | 3 | 4 | 0.97 | 2.43 | 5.69 |
| New | פרפר | butterfly | 4.11 | 2 | 4 | 1.00 | 1.58 | 6.32 |
| New | חתול | cat | 15.66 | 2 | 4 | 0.97 | 2.12 | 6.44 |
| New | מזלג | fork | 2.50 | 2 | 4 | 1.00 | 1.74 | 6.52 |
| New | נבל | harp | 3.47 | 2 | 3 | 1.00 | 2.19 | 6.09 |
| New | סוס | horse | 14.11 | 1 | 3 | 0.94 | 2.06 | 6.26 |
| New | רגל | leg | 56.85 | 2 | 3 | 0.90 | 2.32 | 6.45 |
| New | בואש | skunk | 0.24 | 2 | 4 | 1.00 | 1.94 | 6.15 |
| New | חגורה | belt | 2.98 | 3 | 5 | 0.91 | 1.79 | 6.76 |
| New | חזיר | pig | 8.34 | 2 | 4 | 0.94 | 2.33 | 6.52 |
| New | ספר | book | 343.55 | 2 | 3 | 0.94 | 2.33 | 6.64 |
| New | סנאי | squirrel | 0.56 | 2 | 4 | 0.91 | 2.54 | 6.31 |
| New | נר | candle | 18.19 | 1 | 2 | 0.94 | 1.48 | 6.58 |
